# Cellular lethal damage of ^64^Cu incorporated in mammalian genome evaluated with Monte Carlo methods

**DOI:** 10.3389/fmed.2023.1253746

**Published:** 2023-09-28

**Authors:** Jhonatan Carrasco-Hernandez, José Ramos-Méndez, Elizabeth Padilla-Rodal, Miguel A. Avila-Rodriguez

**Affiliations:** ^1^Departamento de Estructura de la Materia, Instituto de Ciencias Nucleares, Universidad Nacional Autónoma de México, Mexico City, Mexico; ^2^Department of Radiation Oncology, University of California, San Francisco, San Francisco, CA, United States; ^3^Unidad Radiofarmacia-Ciclotrón, Facultad de Medicina, Universidad Nacional Autónoma de México, Mexico City, Mexico

**Keywords:** targeted radionuclide therapy, Auger emitters, DNA, TOPAS-nBio, copper-64

## Abstract

**Purpose:**

Targeted Radionuclide Therapy (TRT) with Auger Emitters (AE) is a technique that allows targeting specific sites on tumor cells using radionuclides. The toxicity of AE is critically dependent on its proximity to the DNA. The aim of this study is to quantify the DNA damage and radiotherapeutic potential of the promising AE radionuclide copper-64 (^64^Cu) incorporated into the DNA of mammalian cells using Monte Carlo track-structure simulations.

**Methods:**

A mammalian cell nucleus model with a diameter of 9.3 μm available in TOPAS-nBio was used. The cellular nucleus consisted of double-helix DNA geometrical model of 2.3 nm diameter surrounded by a hydration shell with a thickness of 0.16 nm, organized in 46 chromosomes giving a total of 6.08 giga base-pairs (DNA density of 14.4 Mbp/μm^3^). The cellular nucleus was irradiated with monoenergetic electrons and radiation emissions from several radionuclides including ^111^In, ^125^I, ^123^I, and ^99m^Tc in addition to ^64^Cu. For monoenergetic electrons, isotropic point sources randomly distributed within the nucleus were modeled. The radionuclides were incorporated in randomly chosen DNA base pairs at two positions near to the central axis of the double-helix DNA model at (1) 0.25 nm off the central axis and (2) at the periphery of the DNA (1.15 nm off the central axis). For all the radionuclides except for ^99m^Tc, the complete physical decay process was explicitly simulated. For ^99m^Tc only total electron spectrum from published data was used. The DNA Double Strand Breaks (DSB) yield per decay from direct and indirect actions were quantified. Results obtained for monoenergetic electrons and radionuclides ^111^In, ^125^I, ^123^I, and ^99m^Tc were compared with measured and calculated data from the literature for verification purposes. The DSB yields per decay incorporated in DNA for ^64^Cu are first reported in this work. The therapeutic effect of ^64^Cu (activity that led 37% cell survival after two cell divisions) was determined in terms of the number of atoms incorporated into the nucleus that would lead to the same DSBs that 100 decays of ^125^I. Simulations were run until a 2% statistical uncertainty (1 standard deviation) was achieved.

**Results:**

The behavior of DSBs as a function of the energy for monoenergetic electrons was consistent with published data, the DSBs increased with the energy until it reached a maximum value near 500 eV followed by a continuous decrement. For ^64^Cu, when incorporated in the genome at evaluated positions (1) and (2), the DSB were 0.171 ± 0.003 and 0.190 ± 0.003 DSB/decay, respectively. The number of initial atoms incorporated into the genome (per cell) for ^64^Cu that would cause a therapeutic effect was estimated as 3,107 ± 28, that corresponds to an initial activity of 47.1 ± 0.4 × 10^−3^ Bq.

**Conclusion:**

Our results showed that TRT with ^64^Cu has comparable therapeutic effects in cells as that of TRT with radionuclides currently used in clinical practice.

## 1. Introduction

**Figure 1:**
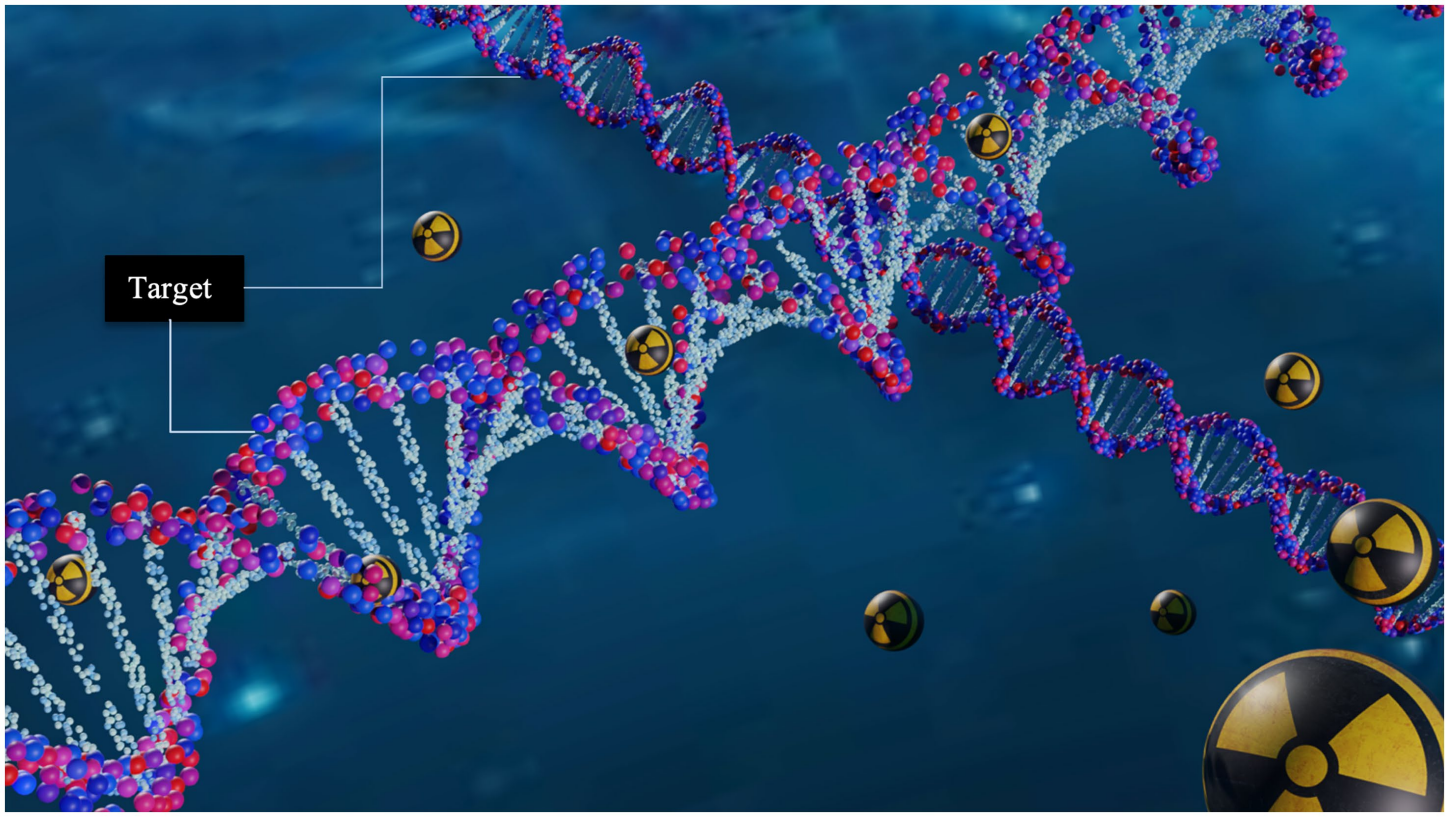
Schematic representation of targeted radionuclide therapy. Its potential to deliver damage with high specificity is due to the capability of the radiopharmaceutical to incorporate the decaying radionuclide near to DNA molecule.

Targeted Radionuclide Therapy (TRT) has shown to be a successful strategy against cancer (1–3). Its success relies on the localized delivery of large amounts of radiation which cause irreversible damage to cancer cells while minimizing the damage to healthy tissue (4). The radiopharmaceuticals used in TRT (Figure 1) consist of a compound (e.g., hormones, peptides, nucleotides, oligonucleotides, and antibodies) and a high-LET emitting radionuclide that specifically binds to a cell site (3).

The most sensitive region to ionizing radiation in the cell is genomic DNA (5). Radiation energy can be deposited in the DNA through direct action -by ionizing charged particles- or indirect action -by interacting with water radiolysis products like hydroxyl radicals, solvated electrons, and hydrogen atoms (6). These interaction processes can lead to two types of DNA damage: a single-strand break (SSB) or a double-strand break (DSB), and in the absence of a DNA repair process, derives in cell death through mitotic catastrophe or apoptosis (7).

Auger emitters (AE) are radionuclides that have aroused a high clinical interest due to their extremely short range, localized dose deposition, and low toxicity when decaying outside the cell nucleus, such as in the cytoplasm (8); examples of AE include ^67^Ga, ^99m^Tc, ^111^In, ^123^I, ^125^I, and ^64^Cu. The AE’s have been shown to have a high relative biological effectiveness, similar to the alpha particles at distances shorter than 11 nm, which is comparable to the DNA molecule’s diameter (8). Auger electrons are ejected from electron orbitals due to nuclear decay modes such as electron capture or internal conversion (9). The energy of those electrons can be greater than 25 keV, but the yield per decay is very low (~ 0.1). Most electrons have energies less than 5 keV and deposit all their energy within a nanometer-micrometer range (9). Furthermore, many of the parent radionuclides also emit β-particles or photons that could be suitable for combined therapy and diagnosis (10).

We can understand the TRT status with AE by analyzing preclinical studies, clinical trials and other novel approaches. In preclinical studies compounds labeled with AE such as [^111^In]In-BnDTPA-F3, [^123^I]MST-312, [^125^I]C5, and [^99m^Tc]C3 have been demonstrated to have a potent cytotoxic effect, intracellular uptake, and DSB induction (11–13). In clinical studies the [^125^I]IUdR and the ^125^I-labeled murine anti-EGFR mAb showed a biological relapse as well as safe and well-tolerated treatments (14). A novel approach using [^111^In]In-DTPA showed no clinical side effects in patients, disease stabilization, and tumor size reduction (14, 15). In addition, over the past decade a new class of radiopharmaceuticals called theranostics have revolutionized nuclear medicine applications. This option opens the possibility of treatment and medical imaging, heralding a new era in the field.

^64^Cu is a radionuclide with theranostics potential that has recently generated broad interest (16), and numerous preclinical reports have explored the therapeutic use of ^64^Cu in experimental mouse models of cancers. For example, Ferrari et al. (17) studied [^64^Cu]CuCl_2_ for glioblastoma 2 (U87MG) in mice, reporting a good response and size reduction in tumors; in some cases, the tumors completely disappeared. Jin et al. (18) evaluated the therapeutic potential of [^64^Cu]Cu-cyclam-RAFT-c(-RGDfK-)4 in glioblastoma cells in mice. Meanwhile, a new type of therapy that combines ^64^Cu -based TRT with immunotherapy has been reached, in order to enhance the therapeutic efficacy of a radiopharmaceutical targeting α_v_β_3_ integrin ([^64^Cu]Cu-DOTA-EB-cRGDfK) (19). On the other hand, Qin et al. (20) demonstrated the therapeutic potential of [^64^Cu]CuCl_2_ for malignant melanoma in mice; the tumor growth was found to be reduced in models that received [^64^Cu]CuCl_2_ treatment. Until recently, only a limited number of clinical studies in humans have been reported using [^64^Cu]CuCl_2_ as radiopharmaceutical, mainly to evaluate the biodistribution and radiation dosimetry in healthy subjects and patients (21, 22).

Various *in vitro* studies have described the DNA-damage inflicted by ^64^Cu. Fernandes-Guerreiro et al. (23) evaluated the radiobiological effects of the [^64^Cu]CuCl_2_ uptake in a panel of PCa cell lines. This study revealed that PCa cells exhibited a higher Cu uptake than non-tumoral cells. Also, they demonstrated that [^64^Cu]CuCl_2_ was able to reach the nuclear cell compartment producing significant genotoxicity and cytotoxicity in PC3, which were less efficient than normal cells in repairing the DNA-damage induced by [^64^Cu]CuCl_2_. McMillan et al. (24) on the other hand performed survival fraction studies with Chinese hamster ovary (CHO) wild type and DNA repair–deficient xrs5 cells exposed to [^64^Cu]Cu-ATSM under hypoxic conditions, and by γH2AX foci assays confirmed DSBs and other complex types of chromosomal aberrations, both typical of high-LET radiation, providing strong evidence that [^64^Cu]Cu-ATSM damages DNA via Auger electrons. More recently, Serban et al. (25) analyzed the DNA-damage and stress responses inflicted in various human normal and tumor cell lines after the exposure to [^64^Cu]CuCl_2_. All investigated cells, regardless of their tumoral or normal status, incorporate ^64^Cu ions similarly, but their fate after exposure was cell-dependent. They found that an activity concentration of 40 MBq/mL of [^64^Cu]CuCl_2_ delivers a therapeutic effect in human colon carcinoma cells, but also caused harm to normal fibroblasts, yet lower than tumoral cells. An activity concentration of 20 MBq/mL was also able to induce DNA-damage and oxidative stress in colon cancer cells, and even when the therapeutic effect on tumor cells might be partial, the radiotoxicity to normal cells is expected to be lower.

Using computational modeling and experiments, researchers have observed and reported DSB caused by AE like ^123^I, ^125^I, ^111^In, and ^99m^Tc when incorporated into the DNA (26–33). We have previously estimated the damage that ^64^Cu, ^125^I and ^111^In caused to the DNA through the use of Geant4-DNA and the DBSCAN algorithm, considering the AE radionuclides randomly distributed in the cellular compartments (such as nucleus, cytoplasm and cell surface); the DNA content was also randomly distributed (no geometrical model) within the nucleus (34). Thus, ^64^Cu has not been studied as a source of DSB damage when it is incorporated into the DNA structure. In the present work, we used a DNA geometry model, incorporated the AE ^64^Cu in two positions within the DNA genome, and calculated the DSB damage as well as the total number of atoms incorporated that would cause a therapeutic effect. The motivation for this research comes from the continuous interest in new radiopharmaceuticals with AE such as the ^64^Cu. Is our hope these data help estimate the total radioactivity needed for treatments against diseases, such as cancer.

## 2. Materials and methods

### 2.1. DNA nuclear model

**Figure 2:**
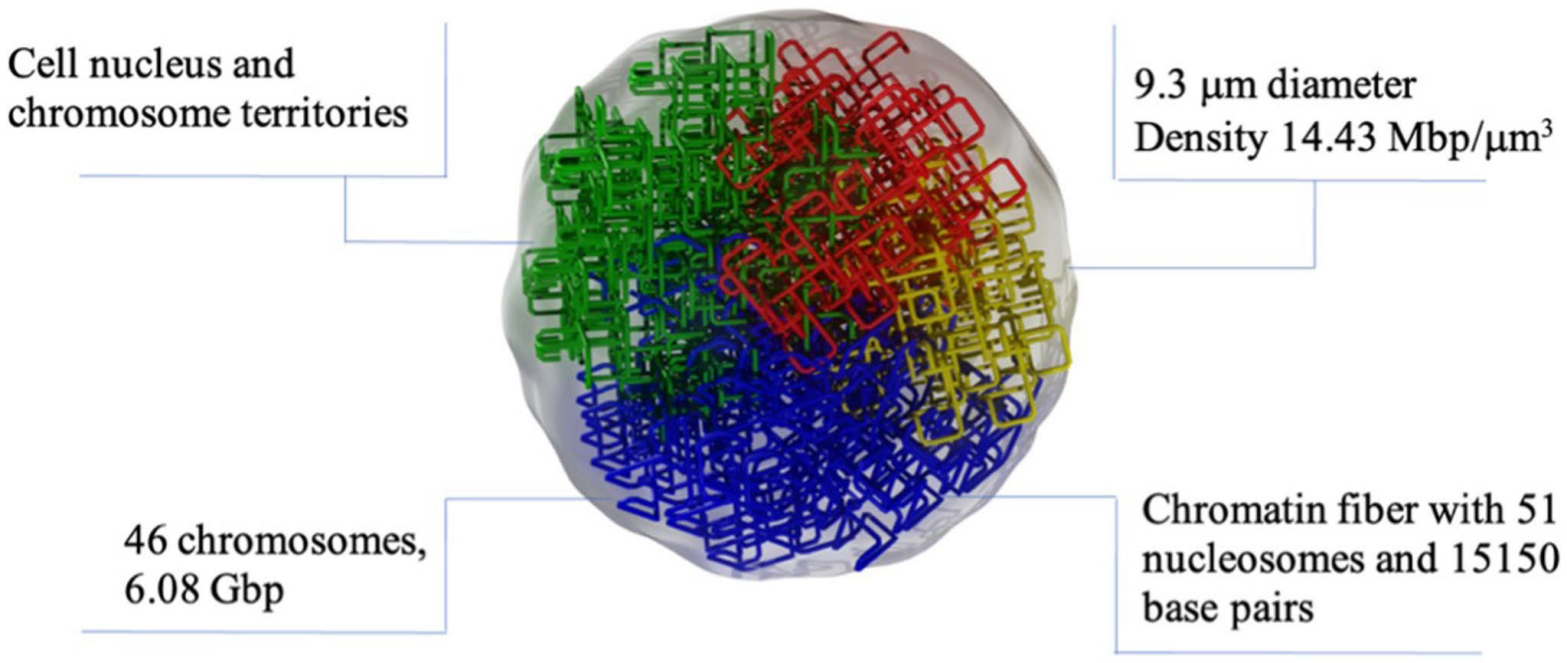
Graphic representation of the nuclear cell model simulated in TOPAS nBio with the typical dimensions and characteristics of mammalian nucleus. The DNA is arranged following a fractal path. Only a few fractal paths are shown in the figure for clarity.

The DNA damage was simulated using TOPAS-nBio (35). TOPAS-nBio is a Monte Carlo track-structure tool built on top of Geant4-DNA (36–38) for modeling the physical, physicochemical, and chemical stages of radiation interactions in liquid water. TOPAS-nBio combines such processes with an extensive library of geometric cell examples and DNA double helix models. We used a mammalian cell nucleus model of 9.3 μm in diameter (Figure 2) that has been previously used to study the cellular response to proton irradiation; the details can be found in Zhu et al. (39). Briefly, Zhu et al. (39), studied the DNA response to a 0.5–500 MeV proton and its repair processes. The direct DNA damage induced by primary and secondary charged particles within the DNA target was modeled through the physics module TsEmDNAPhysics and the chemical interactions of water radiolysis species which were produced in the pre-chemical and chemical stages were modeled with the chemistry module TsEmDNAChemistry. Also, the MEDRAS model (40) was used to describe the DNA damage repair characteristics and chromosome aberration yields. In this work, we focused on estimating the number of DSB.

The spherical nucleus model consists of a DNA double helix configuration which is organized in base pairs, nucleosomes, chromatin fibers and chromosomal structures. The DNA double helix has a diameter of 2.3 nm with a 0.16 nm cylindrical hydration shell surrounding the structure. Each base pair consists of a base, a backbone, and the hydration shell. The bases are represented by half of cylinders of 0.5 nm radius and 0.34 nm thickness, and the backbone is represented as an opposite pair of quarter cylindrical sectors (39). The base pairs are rotated by 36 degrees subsequently. The DNA geometry wraps around a cylindrical histone volume to form the nucleosome; then, multiple nucleosomes form a chromatin fiber. The resulting nucleus consists of 46 chromosomes with a total length of 6.08 giga base-pair (Gbp) of DNA. The cellular nucleus model was placed at the center of a cubic volume (“world”) with a side length of 15 μm.

### 2.2. DNA double strand break scoring

Initial DNA damages within the nucleus, in the form of SSB, may result from either indirect interaction of radiation through radiolytic chemical species with DNA or from direct interaction of radiation with the backbone volume and hydration shell. For modeling indirect damage, the radiolysis products were simulated by Brownian motion step-by-step. Only interactions between hydroxyl radicals (•OH) and the DNA backbone were assumed to induce indirect strand breaks. That means, each time a hydroxyl radical entered a backbone or hydration shell volume, it was removed from the simulation and a SSB was scored with a probability of 0.13. In order to model the direct damage, strand breaks were formed from the physical interactions between the primary and secondary particles, the DNA backbone and hydration shell. Thus, a SSB was scored if at least 17.5 eV of deposited energy was accumulated in a backbone-hydration shell volume.

A DSB was accounted for whenever two SSBs were located on the opposite sides of the DNA double helix, separated by less than 10 base pairs. DSBs were classified into 3 categories depending on their origin: direct DSB, originated from two direct interactions; indirect DSB, originated from two indirect interactions; and hybrid DSB, which comes from one direct interaction and one indirect interaction (41, 42). No classification of clustered DSB was performed in this work.

### 2.3. Irradiation setup

In order to achieve a statistical uncertainty lower than 2% on the DSB yields, the simulations which use monoenergetic electrons and radionuclides required 400,000 and 200,000 statistically independent histories, respectively. The simulations were performed with parallel computing to decrease CPU time, using the Tochtli Cluster built on CentOS 6.8 Linux operating system.

#### 2.3.1. DSB yield verification for monoenergetic electrons

**Figure 3:**
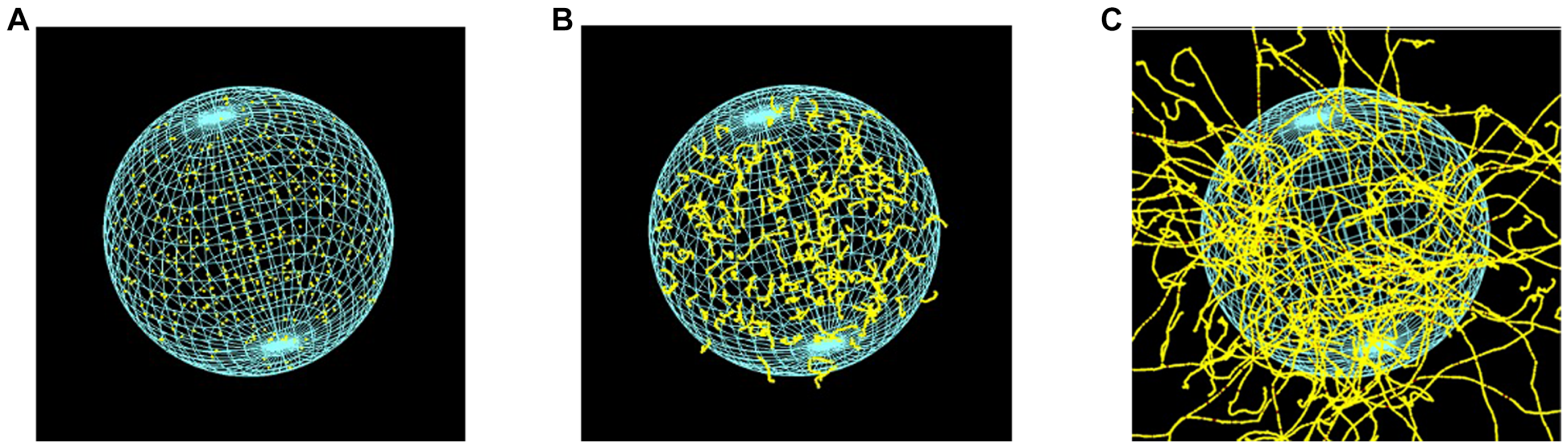
Cellular nuclear model of 9.3 μm in diameter, irradiated by point-like electron sources, randomly distributed inside the cell nucleus, with energies of **(A)** 0.5 keV, **(B)** 5 keV, and **(C)** 20 keV. The visibility of the DNA geometric model was deactivated.

To verify the simulation setup, we calculated the DSB yields produced by the monoenergetic electrons with initial energies within the relevant energy range of AE (43, 44). The energies ranged between 100 eV and 100 keV. The cell irradiation setup consisted of isotropic point sources randomly distributed within the cell nucleus -as illustrated in Figure 3- for electrons of 0.5 keV, 5 keV, and 20 keV. DSB, normalized per dose per Dalton, were compared with the calculated data from Nikjoo et al. (45) and the measured data from De Lara et al. (46) and Frankenberg et al. (47).

#### 2.3.2. Radionuclide incorporated in DNA genome

**Figure 4:**
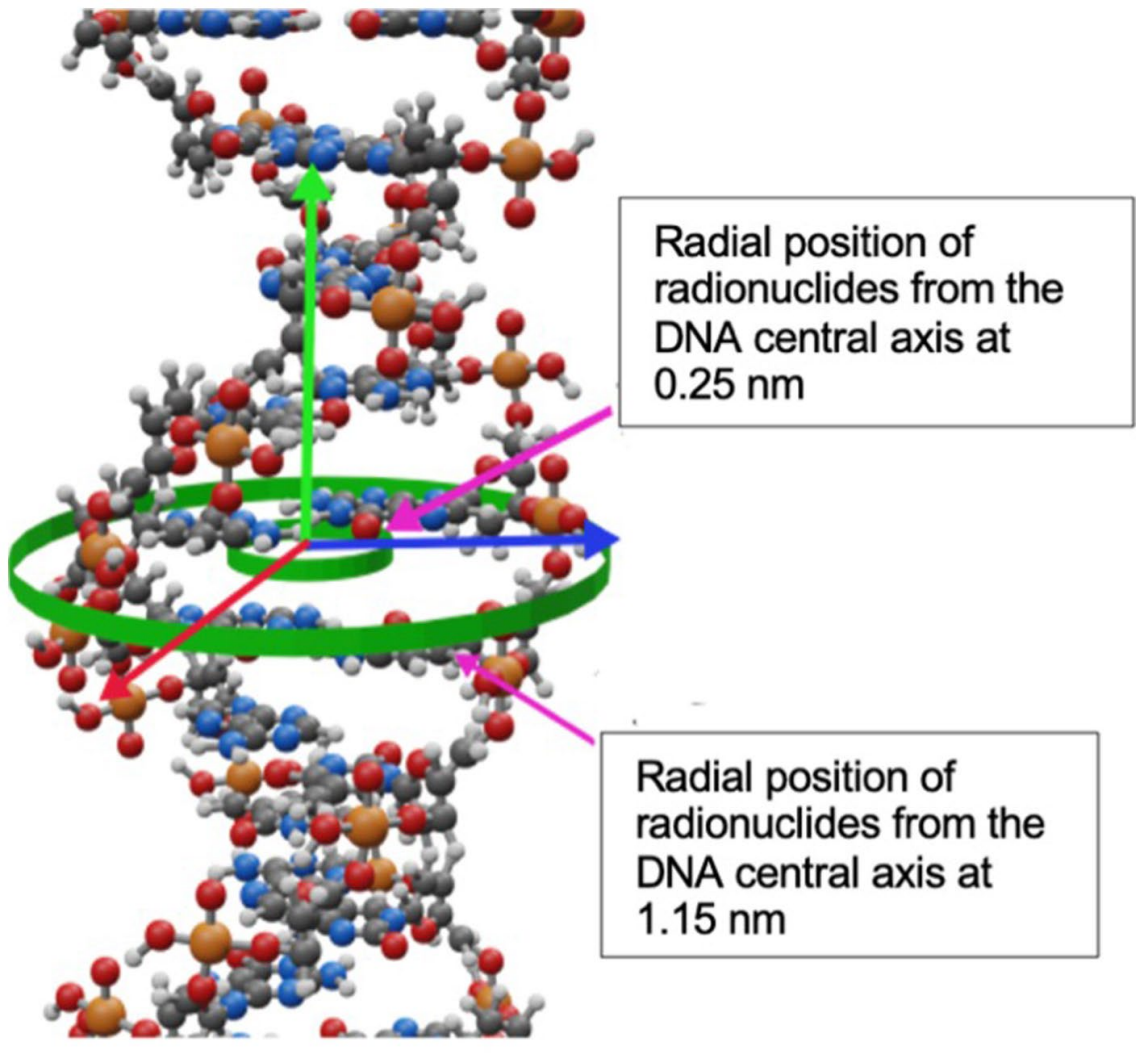
Radial positions of radionuclides from the DNA central axis at 0.25 nm and 1.15 nm.

The internalization of radionuclides into DNA was simulated by placing then radionuclides in one out of two positions along randomly chosen DNA base pairs as it is shown in Figure 4. As depicted, the radionuclides were randomly placed at 0.25 nm or at 1.15 nm off the central DNA axis. The distance between the radionuclide and the central axis of DNA was chosen based on experiments of internalization that are reported in the literature, such as: Goz and Walker (48), who used ^125^I-labeled 5-iodo-2′-deoxyuridine ([^125^I]IUdR) to achieve separation distances shorter than 1 nm between the iodine atom and the DNA central axis. Similarly, Balagurumoorthy et al. (27) reported internalization distances in the 1.046–1.385 nm range for ^123^I and ^111^In, respectively. Karamychev et al. (30) reached a separation distance of 1.13 nm using oligodeoxyribonucleotides (ODNs), and more recently Reissig et al. (32) reported distances between 1.5 nm to 3 nm and the DNA central axis by using ^99m^Tc-labeled pyrene derivatives.

Each radionuclide (^123^I, ^125^I, ^111^In, ^99m^Tc, and ^64^Cu) was simulated for each position configuration in independent simulations. For ^64^Cu, ^123^I, ^125^I, and ^111^In the complete decay, considering all applicable radioactive emissions (i.e., Auger, IC, β+, β-, and photons), was explicitly simulated with the G4RadiactiveDecay module from Geant4, whereas for ^99m^Tc only the total electron spectrum (Auger and IC) obtained from Howell (43) was simulated. DSB were scored and reported as DSB per decay. The radioactive decays in Geant4 are simulated using data libraries from the Evaluated Nuclear Structure Data File (ENSDF) (49).

### 2.4. Initial activity of ^64^Cu to cause lethal damage

The cell susceptibility to lethal damage by ionization radiation is expressed as the D_37_ value (the radiation dose that leads to 37% of cell survival). The lethal damage that ^64^Cu causes in cells was estimated in relation to that from ^125^I. For the ^125^I, Geselowits et al. (50) quantified the toxicity of radiation of the [^125^I]IUdR incorporated in the nucleus of CHO cells in the early S phase. The result was a D_37_ between 40 and 165 decays/cell of ^125^I, which is consistent with the work of Hofer et al. (52) who reported a mean value of ~100 decays/cell. On the other hand, Humm and Charlton (29) derived the following relationship between the total number of DSB (N_DSB_) and the initial number of radioactive atoms (N_0_) attached to DNA base pairs (and hence the activity) which are needed to produce such DSB, as follows:


(1) $$N_{0}=\frac{N_{DSB}}{\left(1-\exp\left(-\lambda t\right))(f+35D\right)}$$

where λ is the decay constant of the radionuclide; *t* is the time for the cell to reach one cell division; *f* is the number of DSB/decay by short-range radioactive emissions (~ nm); and the term 35D accounts for the damage to the genome caused by long-range radiation (~ μm) which is a function of the dose (D) to the nucleus by decay. In this work, we computed N_DSB_ produced by 100 decays/cell of ^125^I as the reference value to quantify cell susceptibility (see Table 1). Thus, to compute N_0_ we substitute N_DSB_ = 194 DSB in Eq. 1, and the term (*f + 35D*) was taken to be equal to the number of DSB/decay obtained for each nuclide in this work, as we are considering both, short- and long-range radioactive emissions. In Eq. 1, the time *t* corresponds to the complete cell cycle from G2 to G1, for which a reasonable value of 24 h is considered. Thus, to account for the first cell division, N_0_ was multiplied by 2 (29).

**Table 1:** DSB per decay for Auger emitters when incorporated at diferent distances from the central DNA axis, including ^64^Cu.

Radionuclide	DSB yield (0.25 nm)	DSB yield (1.15 nm)	Data reported (measured)	Data reported (calculated)
^125^I	1.94 ± 0.01	1.82 ± 0.01	1.01 ± 0.13^a^, 0.82^b^, 0.8^c^, 1.1^i^, 0.52 ± 0.01^i^, 0.24 ± 0.03^i^	2.41 ± 0.8^e^, 1.1 ± 0.01^f^
^123^I	1.20 ± 0.01	1.24 ± 0.01	0.62^b^, 0.74^g^, 0.18 ± 0.01^d^	1.45^e^, 0.62^f^
^111^In	1.09 ± 0.01	1.15 ± 0.02	0.38^c^	0.97 ± 0.38^e^
^99m^Tc	0.378 ± 0.003	0.535 ± 0.001	0.044 ± 0.017^h^	0.86^e^, 0.43^f^
^64^Cu	0.171 ± 0.003	0.190 ± 0.003	–	–

a Krisch and Ley (53).

b Lobachevsky and Martin (31).

c Karamychev et al. (30).

d Balagurumoorthy et al. (26).

e Ftániková and Böhm (28).

f Humm and Charlton (29).

g Makrigiorgos et al. (54).

h Reissig et al. (32).

i Balagurumoorthy et al. (27).

## 3. Results

### 3.1. DSB yields for monoenergetic electrons

**Figure 5:**
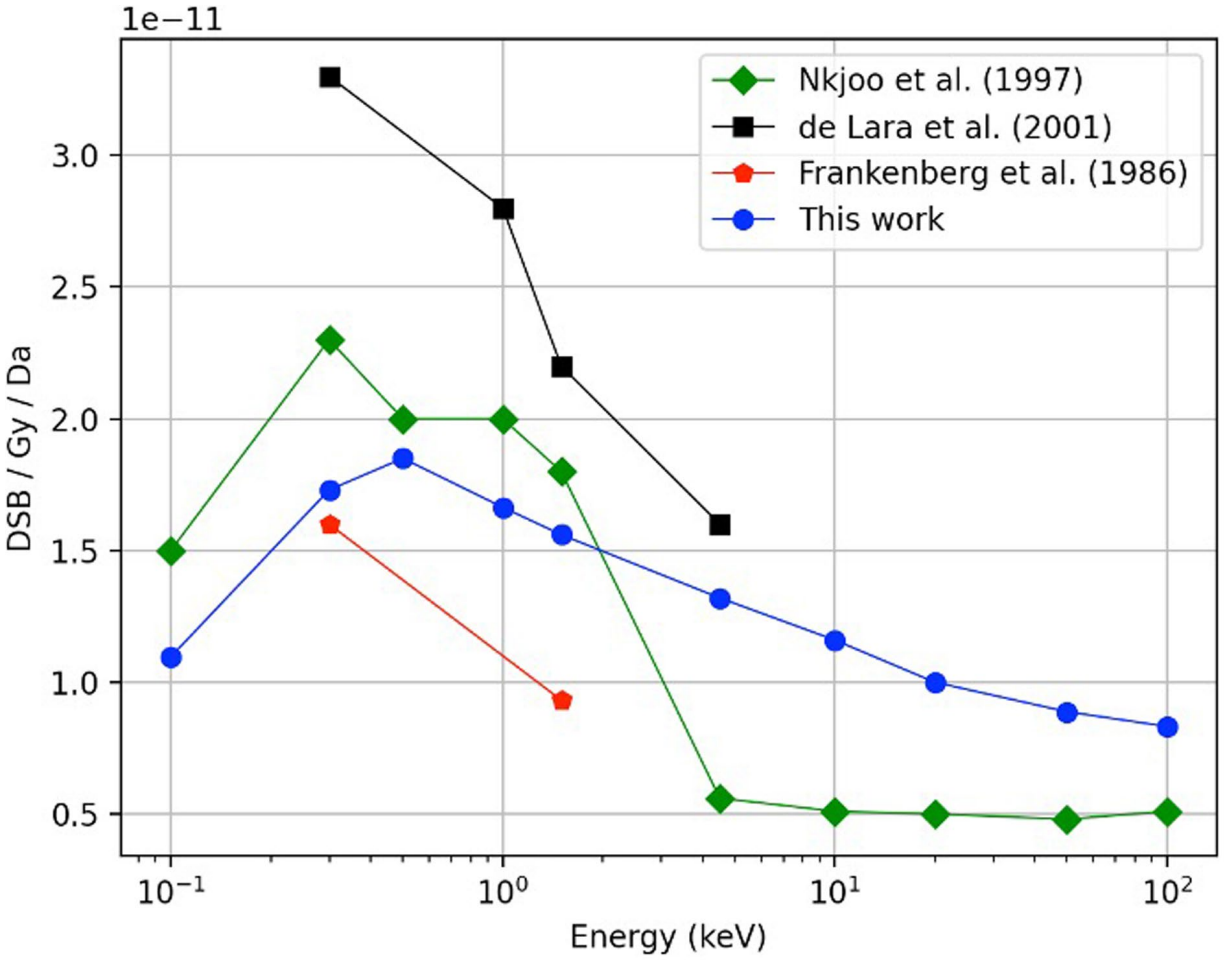
Comparison of the DSB yield as function of electron energy, results obtained in this work (blue circles) with experimental data reported by De Lara et al. (46) (black squares) and Frankenberg et al. (47) (red pentagons). Previously obtained Monte Carlo calculations by Nikjoo et al. (45) using monoenergetic sources of electrons in the energy range of 100 eV to 100 keV are also shown (green diamonds). In this work, a total history number of 400,000 was required for each calculated point.

Figure 5 shows the DSB/Gy/Da for monoenergetic electrons as a function of energy (blue solid circles). As shown, the results exhibit an increasing trend starting at an electron energy of 100 eV (1.10 ± 0.02 DSB/Gy/Da). Later, the curve reaches a maximum value of 1.85 ± 0.03 DSB/Gy/Da at 500 eV. Finally, the DSB yield decreases monotonically until it reaches 0.83 ± 0.01 DSB/Gy/Da at 100 keV. The calculated data from this work follows a similar trend to the calculated data reported by Nikjoo (1997) (51); and falls within the measured data reported by Frankenberg et al. (47) and de Lara et al. (2001) (46).

### 3.2. DSB yields for radionuclides

Table 1 shows the calculated DSB yields for the radionuclides evaluated in this work, including also previously published data. For ^125^I, the DSB/decay decreases from 1.94 ± 0.01 to 1.82 ± 0.01 as the AE’s separation distance relative to the central axis of the DNA increases. This behavior was consistent with the study performed by Balagurumoorthy et al. (27), who reported that the DSB yields decreased from 1.1 to 0.24 DSB/decay for separation distances from the DNA central axis in a range of ~0.7 to 1.39 nm, respectively, which was achieved through [^125^I] IUdR, [^125^I] IEH, and [^125^I]IBH. Conversely, the DSB yields increased with the separation distance for all the other radionuclides.

The yield of the DSB produced by ^64^Cu incorporated in the DNA was determined as 0.171 ± 0.003 DSB/decay for a distance of 0.25 nm from the central DNA axis, and 0.190 ± 0.003 DSB/decay for a distance of 1.15 nm from the central DNA axis. This result is the lowest yield compared with the other radionuclides studied in this work, as shown in Table 1.

### 3.3. Initial activity of ^64^Cu to cause lethal damage

The initial number of atoms to cause lethal damage in a cell of AE relative to ^125^I was calculated for radionuclides localized at the 0.25 nm distance off the DNA central axis (Figure 4). Table 2 compares our results with the reported by Humm and Charlton (29) regarding several radionuclides, including ^64^Cu, and the initial activity per cell (see Section 2.4).

**Table 2:** Comparison of the average number of initial atoms and activities per cell to cause lethal damage when the radionuclide is incorporated at 0.25 nm off the central axis (a random base par).

Radionuclide	Number of atoms incorporated in the genome, Humm and Charlton (29)	Initial activity per cell (Bq × 10^−3^), Humm and Charlton (29)	Number of atoms incorporated in the genome in this work	Initial activity per cell (Bq × 10^−3^) in this work
^125^I	15,650	2.09	17,416 ± 46	2.32 ± 0.01
^123^I	380	5.54	451 ± 2	6.58 ± 0.03
^111^In	–	–	1,625 ± 8	4.65 ± 0.02
^99m^Tc	490	15.67	1,095 ± 4	35.0 ± 0.1
^64^Cu	–	**–**	3,107 ± 28	47.1 ± 0.4

## 4. Discussion

^64^Cu is a very promising AE for TRT due to its favorable nuclear and chemical properties and high cytotoxicity, which is attributed to the generation of low-energy electrons during its decay. Nevertheless, the use of ^64^Cu remains scarce, as there is little evidence of its use on humans to assess its therapeutic potential. Guerreiro et al. (23) reported using a panel of Prostate Cancer (PCa) cell lines exhibiting a deficient DNA damage repair upon exposure to [^64^Cu]CuCl_2_. While Rigui et al. reported a dosimetry study in patients with prostate cancer, showing that the absorbed dose per administered activity was low, they also suggested that clinical trials are needed to evaluate the therapeutic effectiveness of ^64^Cu. Thus, computational modeling is a more convenient for to estimating the therapeutic effectiveness in the short term. To the best of our knowledge, this is the first paper that reports on the DSB yields per decay incorporated in DNA for ^64^Cu.

A comparison study using monoenergetic electrons was performed for model verification purposes. Figure 5 shows the DSB yields for monoenergetic electrons as a function of energy. The DSB yields follow an increasing behavior starting at an electron energy of 100 eV (1.10 ± 0.02 DSB/Gy/Da). The curve reaches a maximum value of 1.85 ± 0.03 DSB/Gy/Da at 500 eV. This behavior is expected because electrons with energy in the range of 100–200 eV have been reported to be two to four times more effective on inducing a DSB than electrons with energy between 100 and 1,000 keV (55–57). Finally, the DSB yield decreases monotonically until it reaches 0.83 ± 0.01 DSB/Gy/Da at 100 keV. While our calculated data fell within the available measured data, the comparison with calculated values reported by Nikjoo et al. (45) show that our results are lower for the energy range from 0.1 to 4.5 keV, but higher for the interval from 4.5 to100 keV. The discrepancies are attributed to the different DNA damage models and cross-section data used from different Monte Carlo engines.

The DSB yield for ^64^Cu incorporated in the genome was simulated by modeling the AE incorporation to 0.25 and 1.15 nm off the DNA central axis and obtaining 0.171 ± 0.003 and 0.190 ± 0.003 per decay, respectively (Table 1).

As a means of verification of the calculation method, DSB yield calculations were performed for the ^125^I incorporated in a DNA base pair, and the results were compared with experimental and calculated data available in the literature for this AE, which is considered the gold standard. Our calculation for ^125^I incorporated in a DNA base pair was 1.94 ± 0.01 (0.25 off the central DNA axis); this value was compared with the data measured by Krisch and Ley (53), who performed studies with ^125^I incorporated into the DNA of bacteriophage in the form of 5-iododeoxyuridine ([^125^I]IUdR) and obtained 1.01 ± 0.13 per decay of ^125^I. Humm and Charlton (29) also obtained a similar value using Monte Carlo simulations. The differences between our result and the reported by Krisch and Ley (53) might be due to the highly packed DNA structure in the cell nucleus model used in this work, which has been shown to be an important factor in the production of DSB’s in comparison with oligonucleotide or plasmid DNA fragments (58). Another study elaborated through Monte Carlo simulation, which was carried out by Ftániková and Böhm (28), obtained a value of 2.41 ± 0.8 DSB per decay for the ^125^I. This calculation is also consistent with Walika’s result, and the differences between our results and the reported by Ftániková and Böhm (28) could be explained because they ignored the protecting character of histones that act as scavengers for chemical species.

The lethality analysis was performed by applying Eq. 1 and the yield of DSB/decay presented in Table 1. The results indicated that the lethality produced by 3,107 ± 28 initial atoms of ^64^Cu incorporated into DNA is equivalent to that of 17,416 ± 46 initial atoms of the gold standard ^125^I in a complete cell cycle of 24 h. This result corresponds to about 0.18 times fewer initial atoms of ^64^Cu to achieve the same lethal damage as ^125^I. The difference in initial atoms to reach the same lethality is mainly due to the longer half-life of ^125^I (60 days) compared with the half-life of ^64^Cu (12.7 h). When we compared with other AE such as ^123^I, ^111^In, and ^99m^Tc we observed that the number of initial atoms was less than the required for ^64^Cu: 451 ± 2, 1,625 ± 8, and 1,095 ± 4, respectively, for the three AE. The differences observed are mainly due to the energies and electron yield per decay of each EA in addition to their half-lives. Table 2 compares our calculation of initial atoms and activities and those reported by Humm and Charlton (29) for the AE ^125^I, ^123^I and ^99m^Tc. The lower values obtained in this work are due to differences in the nuclear cell model, and the DSB yield results from calculations as seen in Table 1.

Regarding the production of ^125^I, this process is carried out mainly in nuclear reactors; however, this technique presents serious disadvantages due to the long hours of irradiation required, and the production of other radionuclides considered contaminants, such as ^126^I with a half-life of 13.1 days. There are other techniques, such as batch production and continuous systems, however, they also present challenges, such as the low amount of useful ^125^I or the need for two irradiation systems in the nuclear reactor’s core (59). On the other hand, many studies have demonstrated the feasibility of the ^64^Cu production through standardized methods in compact cyclotrons and radiosynthesis modules. The impurities produced after the irradiation of the ^64^Cu are extremely low; additionally, they can produce an appropriate quantity and high quality of ^64^Cu, which is suitable for labeling different ligands to be used in therapy and diagnosis (60, 61).

To our knowledge, the lethality of the ^64^Cu upon localization in DNA has not been reported previously. The electron yield (~0.18/decay) during the decay of ^64^Cu is lower in comparison to the other radionuclides evaluated in this work, mainly the ^125^I (~24/decay), which is consistent with the lower number of DSBs produced when it is incorporated in DNA genome. This apparent disadvantage can be offset by the lower number of initial atoms of ^64^Cu needed to produce the same lethality compared to ^125^I (Table 2). On the other hand, most AE must be conjugated to a proper molecule to be incorporated into the vicinity of the DNA and produce cellular toxicity. ^64^Cu has been extensively studied due to its favorable physical and chemical properties with radiolabeled complexes; it has also been successfully evaluated with a wide variety of biomolecules conjugated with suitable chelators with this positron emitter. However, in recent years, ^64^Cu in the simple chemical form of copper dichloride [^64^Cu]CuCl_2_ (without any radiolabeling process) has been identified as a potential agent for TRT because this element is highly regulated naturally at the cellular level through complex molecularly regulated processes that bind and transport copper to different compartments of the cell and cell nuclei, Beaino et al. (62). This fact avoids the need to attach this AE to a specific molecule, thus avoiding the complex process of radiolabeling and the need for expensive target-specific ligands, such as peptides and antibodies, and it would be easy to implement and produce the radiotracer in a suitable form for therapeutic applications. In addition, among the AEs evaluated in this work, apart from ^99m^Tc, ^64^Cu is the only radionuclide that, due to its radioactive emissions during its decay (including positrons), makes it possible to obtain high-quality Positron Emission Tomography (PET) images while producing lethal effects on cells, which provides a considerable advantage.

The main limitation of this study is the estimation of biological effects of the Auger electron emitters incorporated in the DNA structure, without considering the probability of getting inside there. The number of radioactive nuclei reaching a specific molecular target depends on many factors including the affinity of the vector molecule (or radionuclide itself) for the molecular target, the density of the molecular target or specific receptor, and the amount of radioactivity administered. In the specific case of ^64^Cu, experimental studies performed by Fernandes-Guerreiro et al. (23) showed that [^64^Cu]CuCl_2_ is able to reach the nuclear compartment of various PCa cell lines and non-tumoral cells. The percentage of nuclear uptake was cell-dependent and was in the range of 10 to 40%, however it is unknown which percentage of this activity, if any, is incorporated in the DNA structure. Nevertheless, it is possible to consider other approaches to target the DNA structure using molecular vectors such as oligonucleotides, so our assumption of the radioactive nuclei reaching the DNA structure, or its vicinity, is feasible. The next challenge after being capable of binding AEs to DNA in sufficient quantity to cause lethal damage will be to develop suitable methods to estimate the radiation absorbed dose which is an imperative for targeted radionuclide therapy.

## 5. Conclusion

DNA damage caused by ^64^Cu incorporated in the genome was quantified in this work through a nuclear mammalian cell model with Monte Carlo track structure simulations. The therapeutic effect of ^64^Cu, based on the D_37_ value, suggests the ability of this AE to have a lethal effect when incorporated into the DNA genome. The initial activity per cell calculated to cause lethal damage can be used to estimate the total activity necessary to administer in a group of cells or tissue for TRT. On the other hand, although the initial activity of ^64^Cu required to obtain lethality is higher than the required by other AEs analyzed in this work, the number of initial atoms to cause lethal damage is 1/5 times less than the required by ^125^I due to its shorter half- life of 12.7 h for ^64^Cu. Unlike other AE, ^64^Cu emits positrons which allows for PET imaging and provides lethality for cancer cells, making it an excellent candidate for TRT.

Moreover, it is known that copper, due to its chemical properties, is capable of being internalized in cells and nuclei close to DNA without the need to be attached to a vector molecule, unlike other AE radionuclides that need to be attached to molecules to be able to bind to DNA. This characteristic provides a notable advantage, making its production for TRT simpler than other radiopharmaceuticals. However, more studies are needed to understand the molecular processes responsible for its interaction with the DNA molecule and to verify if ^64^Cu, in this form, can bind to DNA in a sufficient quantity to cause lethal damage.

Further studies are required to optimize the subsequent application of ^64^Cu as part of the Targeted Radionuclide Therapy in humans.

## Data availability statement

The original contributions presented in the study are included in the article/supplementary material, further inquiries can be directed to the corresponding author.

## Author contributions

JC-H: Data curation, Formal analysis, Investigation, Methodology, Writing – original draft, Writing – review & editing. JR-M: Conceptualization, Formal analysis, Writing – review & editing. EP-R: Investigation, Methodology, Supervision, Writing – review & editing. MA-R: Conceptualization, Funding acquisition, Resources, Writing – review & editing.
